# Inhibitory circuits in fear memory and fear-related disorders

**DOI:** 10.3389/fncir.2023.1122314

**Published:** 2023-03-23

**Authors:** Sanjay Singh, Lisa Topolnik

**Affiliations:** ^1^Department of Biochemistry, Microbiology and Bio-informatics, Laval University, Quebec City, QC, Canada; ^2^Neuroscience Axis, CRCHUQ, Laval University, Quebec City, QC, Canada

**Keywords:** inhibition, memory, contextual fear conditioning, disinhibition, GABAergic interneuron, optogenetics

## Abstract

Fear learning and memory rely on dynamic interactions between the excitatory and inhibitory neuronal populations that make up the prefrontal cortical, amygdala, and hippocampal circuits. Whereas inhibition of excitatory principal cells (PCs) by GABAergic neurons restrains their excitation, inhibition of GABAergic neurons promotes the excitation of PCs through a process called disinhibition. Specifically, GABAergic interneurons that express parvalbumin (PV+) and somatostatin (SOM+) provide inhibition to different subcellular domains of PCs, whereas those that express the vasoactive intestinal polypeptide (VIP+) facilitate disinhibition of PCs by inhibiting PV+ and SOM+ interneurons. Importantly, although the main connectivity motifs and the underlying network functions of PV+, SOM+, and VIP+ interneurons are replicated across cortical and limbic areas, these inhibitory populations play region-specific roles in fear learning and memory. Here, we provide an overview of the fear processing in the amygdala, hippocampus, and prefrontal cortex based on the evidence obtained in human and animal studies. Moreover, focusing on recent findings obtained using genetically defined imaging and intervention strategies, we discuss the population-specific functions of PV+, SOM+, and VIP+ interneurons in fear circuits. Last, we review current insights that integrate the region-specific inhibitory and disinhibitory network patterns into fear memory acquisition and fear-related disorders.

## Introduction

Fear memories are associated with distressing emotions caused by anticipation of danger. Although essential for survival, when these memories persist even in the absence of threatful cues, it might lead to the development of post-traumatic stress disorder (PTSD). PTSD is a serious mental health condition that promotes reckless and self-destructive behavior due to disturbed activity within neuronal networks (Ford and Courtois, 2014). Initially, Pavlov’s classic fear conditioning paradigm set a benchmark for animal studies aimed at deciphering the behavioral and neural circuits involved in fear learning (Pavlov, 1927). Since then, rodent models of fear conditioning have been extensively used in laboratory studies to understand the physiological processes that make animals more alert to traumatic events and lead to the development of associative memory. In this paradigm, the animals are trained to receive a neutral conditioning stimulus (e.g., auditory tone: CS+) paired with an aversive stimulus (mild foot shock: US) in a specific environmental context. Another neutral stimulus remains unpaired (CS-). After the repeated presentation of CS+US pairing (generally 4–5 trials), the animals learn to associate the CS+ with the US, which is referred to as fear conditioning (Rescorla, 1988). As a result, the presentation of the CS+ evokes the fear response manifested in the form of freezing or the inhibition of motor activity. Accordingly, the aversive stimuli can be considered as a dominating cue that can elicit memory formation and even change the individual’s behavior. To overcome the altered behavioral response, the extinction paradigm is routinely used by neuroscientists (Myers et al., 2006). Extinction can be defined as a form of learning, in which animals learn to inhibit retrieval (Konorski, 1948, 1967). In this paradigm, the CS+ (tone in the case of cued fear learning or context in the case of contextual fear learning) is repeatedly presented in the absence of the US leading to the reduction of fear response either by the erasure of the originally acquired CS+US association or the formation of a new association (Quirk et al., 2010). This paradigm allows for attenuating the reactivated fear memories by disrupting reconsolidation and is used in the therapy of PTSD and other related disorders.

Several decades of work have established that the experience-dependent plasticity induced by the CS-US association underlies the development of intrusive memories and causes alterations in specific brain circuits and behavior (Franke et al., 2021). To understand the circuitry involved in fear memory processes, neuroscientists have focused primarily on the triad of brain regions that includes the amygdala, hippocampus, and medial prefrontal cortex (Feng et al., 2014; McEwen et al., 2016; Kredlow et al., 2021). The amygdala is considered as the key brain region involved in the regulation of emotional responses (Phelps and LeDoux, 2005; Pape and Pare, 2010; Forster et al., 2017). This almond-shaped structure located in the medial temporal lobe is composed of >10 nuclei, with the basolateral complex (BLA) and the central amygdala (CeA, comprising the centro-lateral (CeL) and the centro-medial (CeM) regions) being intensively studied in the context of fear memory. It has been established that both acquisition and consolidation of fear memory rely on synaptic plasticity and protein synthesis in the amygdala (Schafe et al., 1999; Maren et al., 2003). The hippocampus is a second important region of the limbic system that is mainly involved in contextual (Raineki et al., 2010; Oh and Han, 2020) and traces fear learning, which relies on a temporal association between the CS and US (Curzon et al., 2009; Sharma et al., 2018). Hippocampus makes a part of the hippocampal formation comprising the hippocampus proper that consists in the CA1, CA2, and CA3 areas, the entorhinal cortex, the dentate gyrus (DG), and the subiculum (Wright, 2020). The hippocampal CA1, CA3, and DG areas have been shown to play a crucial role in contextual fear memory encoding, consolidation, and retrieval (Maren et al., 1997; Sierra-Mercado et al., 2011; Liu et al., 2012; Donato et al., 2013; Ramirez et al., 2013; Lovett-Barron et al., 2014; Stefanelli et al., 2016). Another vital region that participates in the regulation of the fear response is the prefrontal cortex (PFC), with the prelimbic area (PL-PFC) involved in the expression of fear memory, and the infralimbic area (IL-PFC) implicated in the extinction memory (Quirk et al., 2000; Runyan et al., 2004; Laviolette et al., 2005). Whereas the critical role of these brain regions in fear memory has been established, previous research focused mainly on excitatory neurons; however, with advancements in this field, it has become clear that, in addition to excitatory connections, inhibitory inputs are equally important for fear memory acquisition, consolidation, and extinction (Letzkus et al., 2011; Donato et al., 2013; Lovett-Barron et al., 2014; Stefanelli et al., 2016; Tipps et al., 2018; Krabbe et al., 2019).

Across different cortical and limbic structures, the inhibitory inputs are delivered by the GABAergic inhibitory neurons (or interneurons) that provide spatio-temporal coordination of the activity of principal cells (PCs) via the inhibition of specific subcellular domains of PCs, such as soma and dendrites (Klausberger and Somogyi, 2008; Capogna, 2014; Tremblay et al., 2016; Pelkey et al., 2017). In addition, activation of inhibitory interneurons can result in circuit disinhibition, or reduction in inhibition of PCs, because of extensive connectivity between interneurons (Cummings and Clem, 2020; Kullander and Topolnik, 2021). GABAergic neurons are highly heterogeneous and comprise a large set of specific cell types with distinct morphological, physiological, molecular, and functional properties (Ascoli et al., 2008; Klausberger and Somogyi, 2008; Capogna, 2014; Tremblay et al., 2016; Pelkey et al., 2017). Understanding the cell-type-specific roles of GABAergic neurons in different brain areas remains therefore a challenging task. Nonetheless, the development of genetic intervention strategies allowed for the selective targeting of three populations of interneurons that account for most GABAergic cells in cortical and limbic areas, i.e., cells expressing the calcium-binding protein parvalbumin (PV+), the neuropeptide somatostatin (SOM+), or the vasoactive intestinal peptide (VIP+; Rudy et al., 2011; Capogna, 2014; Kepecs and Fishell, 2014). This article will first review the evidence from human studies regarding the major brain structures involved in fear memory and fear-related disorders, such as the amygdala, hippocampus, and the prefrontal cortex, and then discuss the animal studies that allowed deciphering the circuit and cellular mechanisms underlying fear memory acquisition and extinction. As more recent research has explored the specific contributions of inhibitory interneurons in different types of memory (Topolnik and Tamboli, 2022), in the sections dealing with the related circuits, the attention will be focused on populations of PV+, SOM+, and VIP+ interneurons in order to understand their specific functions in modulating the cortical and limbic circuitry involved in fear learning.

## Major brain structures involved in fear memory and related disorders: evidence from human studies

It has been established that the amygdala, PFC, and hippocampus are all involved in threat learning and are affected in individuals with PTSD (Bremner, 2006), with each of the structures playing its own role in relation to fear expression and extinction. First, studies conducted on patients with unilateral or bilateral amygdala lesions revealed impaired fear learning (Bechara et al., 1995; LaBar et al., 1995; Weike et al., 2005). Similarly, war veterans with the damaged amygdala exhibited reduced fear responses (Koenigs et al., 2008), indicating that the amygdala is critical for the physiological expression of fear learning in humans. Moreover, the amygdala showed consistent alterations in morphology and activity in fear-induced disorders. The structural magnetic resonance imaging (MRI) conducted on patients with PTSD diagnosis showed a reduced volume of the amygdala (Morey et al., 2012; Zhang et al., 2021). The smaller amygdala volume could also make the individual more prone to the development of PTSD (Morey et al., 2012). Furthermore, the functional neuroimaging studies also demonstrated that, during fear learning in humans, the CS+ presentation resulted in high activation of the amygdala (LaBar et al., 1998; Sehlmeyer et al., 2009). Such studies also showed that the fear memory consolidation and extinction are both associated with memory traces in the BLA (Bach et al., 2011; Agren et al., 2012; Björkstrand et al., 2015). In addition, the prefrontal control of the amygdala important for fear expression and extinction can be impaired in individuals with PTSD (Forster et al., 2017). A more recent study using simultaneous positron emission tomography and functional MRI revealed an additional link between the amygdala and striatum by showing that, during conditioning, activity in the amygdala is facilitated by dopamine release, which can control the strength of conditioned fear response (Frick et al., 2021). Therefore, the amygdala is a central component in the brain threat circuitry critical for the acquisition and consolidation of fear memories and the expression of fear-related disorders.

Second, like the amygdala, the hippocampus also showed altered morphology in fear-related disorders. The reduction in hippocampal volume was observed in patients suffering from PTSD and untreated depression (Sheline et al., 2003; Kitayama et al., 2005; Zhang et al., 2021). Both the left and right hippocampi exhibited reduced volume, with case-specific variability (Pavić et al., 2007; Nelson and Tumpap, 2017). In addition to structural changes, the PTSD patients showed altered hippocampal activity, although the findings are contrasting. Both reduced (Etkin and Wager, 2007; Hayes et al., 2011) and increased hippocampal activity (Shin et al., 2004) have been observed in patients with PTSD diagnosis, likely because of the patient history-/treatment-related specifics of the examined cases. Whereas the results are contradictory, they still suggest that the hippocampus is involved in the fear response in humans. In healthy individuals, the hippocampus has been also shown to gate the extinction memory (Sevinc et al., 2019). Interestingly, various models of PTSD have revealed a compromised GABAergic inhibition in the hippocampus, which results in symptoms that are consistent with dysregulation of affective control and the extinction of conditioned fear. For example, using ^1^H magnetic resonance spectroscopy, it has been shown that suppression of unwanted thoughts was associated with higher hippocampal GABA concentrations and a stronger fronto-hippocampal coupling, pointing to an important role of hippocampal interneurons in regulating fear extinction (Schmitz et al., 2017). Further experimental evidence will be required to understand the exact role of the human hippocampus in fear memory acquisition and extinction.

Third, different neocortical areas, including the prefrontal, insular, temporal, parietal, and occipital regions, also displayed abnormal volume in individuals with PTSD (Wang et al., 2021). The reduced volume of the ventromedial prefrontal cortex (vmPFC) is also reported in patients suffering from stress-related disorders (Greco and Liberzon, 2016). Moreover, the cued delayed and trace fear conditioning triggered an increase in the activation of the anterior cingulate and insular cortices during fear memory acquisition (Sehlmeyer et al., 2009). Further, the repetitive transcranial magnetic stimulation of the dorsal prefrontal cortex (dlPFC) during the reconsolidation window resulted in reduced fear expression and fear return after extinction (Borgomaneri et al., 2020). In a recent study conducted by Anderson and Floresco (2021), the role of prefrontal regions was investigated in the retrieval-stopping test, an assay where human volunteers were allowed to terminate the retrieval of fear memory, and significant activation was detected in the dlPFC, ventrolateral prefrontal cortex (vlPFC), posterior middle frontal gyrus (pmFG), and bilateral insular cortex. Among these regions, the dlPFC was extensively activated. The vmPFC in turn was activated during late fear conditioning (Fullana et al., 2016) and extinction learning (Phelps et al., 2004), whereas bilateral lesions in this region were associated with impaired fear conditioning (Battaglia et al., 2020). Collectively, these lines of evidence from human studies highlight the fear-induced structural and functional alterations within three primary brain regions comprising the fear network, such as amygdala, hippocampus, and the prefrontal cortex, which together regulate the fear-induced adaptive behavior but are compromised in fear-related disorders. A deeper understanding of the circuit and cellular mechanisms underlying these processes has been provided in animal studies.

## Animal studies: amygdala

Similar to human studies, changes in the structure and function of the amygdala have been linked with fear learning in animal models. In particular, it has been shown that the BLA complex of the amygdala is playing a key role in fear memory (Marek et al., 2019; Ponserre et al., 2022). As such, during auditory fear learning, subjects learn an association between a tone and a mild electric foot shock. The BLA receives two streams of inputs regarding auditory (CS, tone) and somatosensory information (US, foot shock; Ponserre et al., 2022). Through the repeated temporal association of these inputs, the subjects exhibit defensive responses to the CS alone [i.e., conditioned response (CR)], which is driven by the amygdala’s response to the auditory CS information entering through the BLA and going to the CeL, CeM and to the periaqueductal gray (PAG; Dejean et al., 2015; Tovote et al., 2015). The BLA model of fear conditioning suggests that the integration of CS and US information by the BLA PCs leads to long-term potentiation (LTP) at the synapses that carry the auditory CS (Schafe et al., 1999; Pape and Pare, 2010); although the auditory cortex and thalamus are also involved in fear conditioning-induced plasticity (Letzkus et al., 2011). Furthermore, blockade of the LTP in BLA using a protein synthesis inhibitor or manipulation with MAPK activity and PKA impaired fear memory consolidation. In fact, both long-term memories for contextual fear learning and auditory fear conditioning were affected, while no effect on short-term memory was observed (Atkins et al., 1998; Schafe et al., 1999). In addition to the induction of synaptic plasticity, other processes that combine enhanced intrinsic excitability and changes in the recruitment of inhibitory interneurons may occur in parallel (Han et al., 2007; Kim et al., 2013). Together, these intrinsic, synaptic, and circuit mechanisms will be responsible for assignment of some LA PCs to fear memory ensembles.

**Figure 1:**
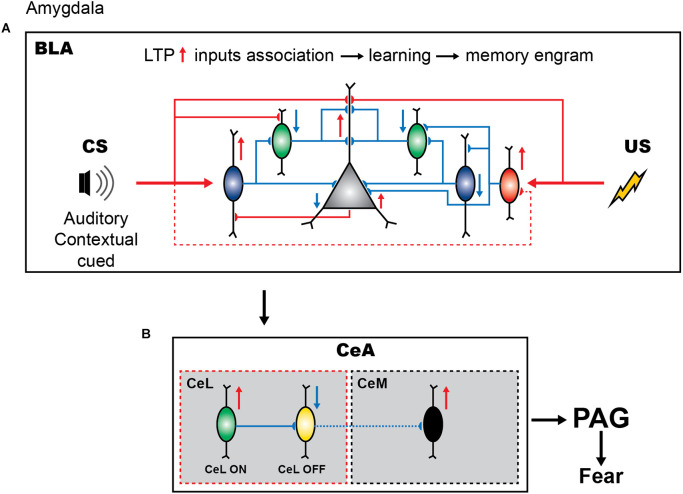
Inhibitory circuits of amygdala in fear learning. **(A)** Presentation of neutral stimulus (CS) elicits the firing of PV+ interneurons (blue) and causes the dendritic disinhibition of principal cells by inhibiting SOM+ cells (green), thus promoting the sensory response and enabling the tone-shock association. In contrast, US presentation results in the dendritic and perisomatic disinhibition due to inhibition of SOM+ and PV+ cells by the upstream VIP+ interneurons (red). **(B)** The cue-shock associated information is projected to the CeL and causes the activation of the SOM+ expressing CeL neurons (green). These GABAeric neurons regulate the CeM-PAG pathway for the expression of fear by inhibiting the PKC-delta expressing CeL cells.

Importantly, different stages of amygdala-dependent learning can be tightly controlled by synaptic inhibition mediated by the BLA GABAergic neurons (Figure 1A; Shumyatsky et al., 2002; Szinyei et al., 2007; Wolff et al., 2014; Krabbe et al., 2019). Several interneuron subtypes have been identified in the BLA based on cytosolic markers and physiological properties (reviewed in Spampanato et al., 2011; Hajos, 2021). Among others, the PV+, SOM+, and VIP+ populations are all involved in fear learning, albeit via distinct circuit mechanisms (Figure 1A; Wolff et al., 2014; Krabbe et al., 2019). Similar to cortical areas, PV+ interneurons, which comprise basket and axo-axonic cells, represent one of the largest inhibitory populations in the BLA and provide inhibitory inputs to PCs and GABAergic cells, including SOM+ interneurons (McDonald and Mascagni, 2002; Woodruff and Sah, 2007a,b; Wolff et al., 2014; Bocchio et al., 2015; Vereczki et al., 2016, 2021). BLA PV+ interneurons are primarily driven by PCs (Smith et al., 2000), and play an important role in synchronizing PC firing through feedback perisomatic inhibition (Woodruff and Sah, 2007a,b; Bienvenu et al., 2012; Veres et al., 2014, 2017). Single-unit recordings *in vivo* from opto-tagged PV+ interneurons revealed that the majority of PV+ cells are excited by CS and inhibited by shock presentation (Wolff et al., 2014; although the axo-axonic cells may be activated by the shock or hind paw pinches, see Bienvenu et al., 2012). Moreover, additional optogenetic activation of PV+ cells during fear conditioning attenuated the CS freezing response, whereas their inhibition during the US led to increased freezing during fear memory retrieval. These data indicate that the CS-induced activation and US-induced inactivation of PV+ cells are required for fear memory acquisition. Furthermore, PV+ interneurons have been shown to control the memory engram size in the BLA (Morrison et al., 2016), which may rely on the input- and/or target-specific plasticity rules at either excitatory or inhibitory connections, and require further investigation.

Local SOM+ interneurons in the BLA are primarily driven by cortical inputs providing feedforward inhibition to the distal dendrites of PCs (Figure 1A; Muller et al., 2007; Unal et al., 2014; Wolff et al., 2014). These cells receive inhibitory inputs from the PV+ basket cells and VIP+ interneurons (Wolff et al., 2014; Krabbe et al., 2019). Similar to the cortical SOM+ population, the BLA SOM+ family may comprise a variety of cell types with distinct functions (McDonald and Mascagni, 2002; Yu et al., 2016). For example, some SOM and neuropeptide Y (NPY) co-expressing BLA interneurons exhibit properties of cortical neurogliaform cells with slow GABA signaling (Mańko et al., 2012) that can shape hippocampo-amygdala interactions during fear memory retrieval (Seidenbecher et al., 2003). Also, some long-range GABAergic projections arising from the BLA interneurons that co-express SOM and NPY have been reported (McDonald et al., 2012). Whereas their cell-type-specific functions remain to be determined, genetically targeted manipulations at the population level revealed an important role of SOM+ interneurons in fear memory. In particular, using the combination of optogenetic manipulations and single-unit recordings, Wolff et al. (2014) reported that, during the auditory CS presentation, the activity of SOM+ interneurons is suppressed in line with a remarkable increase in PV+ cell activity. Furthermore, the US presentation also caused the inhibition of SOM+ interneurons. Driving the SOM+ interneuron activity during the CS presentation resulted in reduced learning, whereas the inhibition of these cells led to increased learning. These data indicate that, contrary to PV+ interneurons, inhibition of SOM+ cells is important for fear memory acquisition. Thus, whereas both PV+ and SOM+ interneurons are involved in fear learning, they have opposite functions (Figure 1A). During CS presentation, the firing of PV+ interneurons can result in perisomatic inhibition, followed by the dendritic disinhibition of PCs via the inhibition of SOM+ cells. During US presentation, the inhibition of both the PV+ and SOM+ interneurons will result in the perisomatic and dendritic disinhibition of the PCs, respectively. Together these inhibitory and disinhibitory circuit motifs may be required to synchronize PC ensembles, support the CS-US association, and gate fear memory acquisition.

VIP+ interneurons in the BLA form a heterogeneous population and target both the PCs and interneurons, such as PV+, SOM+, cholecystokinin-expressing (CCK+), and neurogliaform cells, as well as VIP+ cells, providing synaptic inhibition with target-specific properties (Rhomberg et al., 2018; Krabbe et al., 2019). Thus, VIP+ interneurons are involved in both the inhibitory and disinhibitory circuits. Recent elegant work, using genetically targeted calcium imaging *in vivo*, revealed that the firing of BLA VIP+ interneurons is greatly enhanced during the presentation of the US, and is primarily driven by the acetylcholine released from the basal forebrain afferents (Krabbe et al., 2019). VIP+ cells have been also recruited by the CS alone but at a lower fraction as compared to the US alone or CS-US pairing. Accordingly, optogenetic inhibition of VIP+ interneurons during the US presentation prevented fear memory formation (Krabbe et al., 2019). Thus, it can be suggested that, via the target-specific inhibition of both the PV+ and SOM+ interneurons, VIP+ cells can select PV+ interneurons that will be active during conditioning and together with PV+ cells contribute to SOM+ silencing, providing a higher level regulation over inhibitory circuits required for associative fear learning.

The CeA GABAergic cells are also involved in fear encoding and defensive/aversive behaviors (McDonald and Augustine, 1993; Paré and Smith, 1993). In fact, after CS-US association in the BLA, the information is received by the CeL, which in turn regulates the CeM-to-PAG projecting neurons during the subsequent presentation of CS neutral stimulus (Figure 1B; Dejean et al., 2015). The CeL comprises two populations of interneurons that show excitatory and inhibitory CS responses after fear conditioning (Figure 1B; Ciocchi et al., 2010; Duvarci et al., 2011). As a result, with the presentation of CS, CeL SOM+ neurons (Li et al., 2013; Penzo et al., 2015; Yu et al., 2016) become activated and, *via* the inhibition of the protein kinase C delta (PKC-δ)-expressing neurons projecting to the CeM, cause disinhibition of the CeM-to-PAG pathway, leading to the expression of the fear response by the PAG (Ciocchi et al., 2010; Li et al., 2013; Dejean et al., 2015). Indeed, the inactivation of CeM neurons using a fluorescently labeled GABA_A_ receptor agonist muscimol-bodipy impaired the freezing behavior, while optogenetic activation of CeM neurons elicited freezing (Ciocchi et al., 2010). It should be noted, however, that the CeA GABAergic population may consist of different interneuron types with specific connectivity motifs that may play an additional role in regulating the CeL-to-CeM pathway during fear conditioning; the cellular diversity of the CeA inhibitory population remains largely unstudied. For example, a population of SOM+ CeL neurons exhibited potentiated LA synapses during fear conditioning. Furthermore, optogenetic activation of these cells elicited freezing behavior whereas their silencing impaired fear learning (Li et al., 2013). These cells do not project to CeM and it is currently unknown whether they form local inhibitory or disinhibitory microcircuits with CeL-_ON_ neurons to modulate their activity.

Furthermore, both BLA and CeA inhibitory circuits may be important for fear extinction (Herry et al., 2008; Duvarci et al., 2011). Enhanced inhibition can suppress fear expression by reducing the activation of “fear neurons” that exhibit a pronounced response following fear conditioning (Herry et al., 2008). In fact, an overall increase in GABAergic inhibition has been observed in the BLA after extinction training (Chhatwal et al., 2005; Heldt and Ressler, 2007). Interestingly, the PV+ interneuron synapses formed onto BLA PCs undergo long-term potentiation during contextual fear extinction, thus representing an important mechanism for silencing the “fear neurons” (Herry et al., 2008; Trouche et al., 2013). Alternatively, local disinhibitory processes may allow for the formation of the new memory traces or extinction ensembles in the BLA. Hence, it would be important to determine the relative weight of different GABAergic elements that form inhibitory and disinhibitory patterns during fear extinction. Furthermore, a recent sophisticated study combining *in vivo* calcium imaging with functional manipulations revealed a critical role of the intercalated (ITC) clusters in orchestrating the transitions between the high- and low-fear states and fear extinction (Hagihara et al., 2021). The ITC clusters are located between BLA and CeA and are populated by GABAergic neurons that comprise a mutually connected inhibitory network to control the amygdala output pathways in response to changes in the environment. The results of this work thus indicate that the balance in the activity of ITC circuits may control a wide range of amygdala functions and adaptive behavior.

## Animal studies: hippocampus

Contextual fear conditioning (CFC) has been considered as a robust behavioral paradigm to investigate the role of the hippocampus in fear learning (Kim and Fanselow, 1992; Raineki et al., 2010; Liu et al., 2012; Kim and Bin, 2020; Oh and Han, 2020). During CFC, animals develop an associative memory between the neutral environmental context and the aversive stimulus, which prompts them to adopt a defensive behavior upon re-exposure to the conditioning context. The CFC paradigm combines two stages: first, the animal collects unified multisensory environmental context information; second, it associates the environmental context information with the aversive stimulus (Fanselow, 1986; Fanselow and Poulos, 2004). It has been established that the hippocampus integrates the multisensory features of the environment into a representation of context; however, it must also exclude sensory features about aversive stimulus (Fanselow et al., 1993).

Different parts of the hippocampus appear to play different functions in these processes. The dorsal hippocampus (dHPC) is primarily involved in spatial learning and episodic memory, whereas the ventral hippocampus (vHPC) receives inputs from the amygdala and the hypothalamus, and is involved in the regulation of stress and emotional responses (Fanselow and Dong, 2010). In rodent studies, the presentation to the animals of the mild electric foot shock in a neutral context in the absence of a tone triggers the development of associative fear memory. This paradigm has been widely used for studying contextual fear memory encoding and consolidation (Curzon et al., 2009). Using this paradigm, it has been established that the CS is encoded by the dHPC, the output of which is subsequently associated with the US through synaptic plasticity in the amygdala (Kim and Fanselow, 1992). Furthermore, the role of dHPC in fear encoding was confirmed by using lesions-based studies. A lesion in the dHPC on the day after CFC greatly diminished learning, whereas lesions performed 30–100 days after fear conditioning yielded minimal disruption in the acquisition of contextual fear learning. In contrast, during extinction training, conditioned animals are only exposed to the same context in which conditioning was performed, without any shock (Curzon et al., 2009). Rosas-Vidal et al. (2014) demonstrated that the activation of the vHPC during extinction training resulted in the upregulation of the brain-derived neurotrophic factor and suggested that the vHPC, *via* connections with the IL-PFC, is involved in fear memory extinction.

Whereas the role of the amygdala in fear acquisition and extinction mechanisms as well as in interactions with other brain areas have been extensively studied, relatively little is known about the contribution of hippocampal circuitry. The primary output neurons of the hippocampus, the PCs in area CA1, are driven by the Schaffer collateral pathway from the CA3 region and the temporo-ammonic pathway from the entorhinal cortex (Ahmed and Mehta, 2009). Whereas the CA3 region is responsible for a unified representation of a multisensory context, the entorhinal cortex conveys discrete sensory information in relation to the context (Maren et al., 1997; Kesner, 2007). Electrolytic lesions of the fimbria/fornix, dHPC, or entorhinal cortex produced anterograde deficits in CFC in rats (Maren et al., 1997). At the cellular level, nonlinear interactions between the CA3 and the entorhinal cortex inputs in dendrites of PCs can result in burst-spiking output and synaptic plasticity (Golding et al., 2002; Takahashi and Magee, 2009). Although PCs can carry behaviorally relevant information in the timing of spikes (Jones and Wilson, 2005), spike rate (Ahmed and Mehta, 2009), and spike bursts (Harris et al., 2001), the information conveyed using the bursts of spikes alone is sufficient for the encoding of context in the hippocampus during fear learning (Xu et al., 2012). Importantly, the pharmacological delivery of muscimol (an agonist of the GABA-A receptor) to the dHPC prior to fear conditioning caused impairment in contextual fear memory encoding, highlighting the critical role of hippocampal interneurons in the regulation of fear acquisition (Oh and Han, 2020). Hippocampal interneurons are largely heterogeneous and exhibit distinct morphological, neurochemical, transcriptomic, and physiological properties (Klausberger and Somogyi, 2008; Pelkey et al., 2017; Harris et al., 2018). Specifically, in the hippocampal CA1 area, spike timing of PYRs is primarily regulated by PV+ interneurons that inhibit the perisomatic region of PCs, whereas burst spiking is regulated by SOM+ interneurons that inhibit PC dendrites (Losonczy et al., 2010; Royer et al., 2012). This functional dissociation between CA1 SOM+ and PV+ interneurons suggests that these cells may play distinct mnemonic functions, with SOM+ interneurons being a primary candidate for regulation of associative learning.

**Figure 2:**
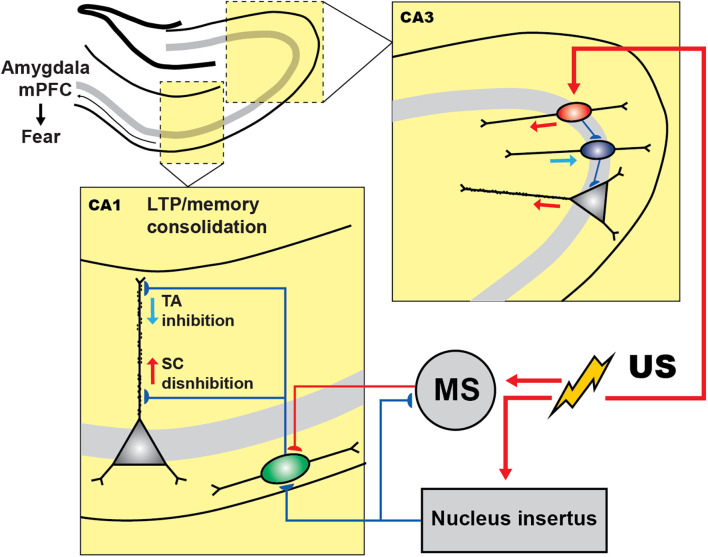
Inhibitory and disinhibitory circuit motifs in the hippocampus during fear encoding. In CA3 region of hippocampus shock presentation induces the PC disinhibition *via* inhibiting the PV+ cells (blue) by the activation of VIP+ population (red), which can control the fear expression. In CA1, during CFC, shock presentation resulted in the activation of the medial septum (MS) whose excitatory inputs to SOM+ cells (green) cause the PCs dendritic inhibition. However, the activity of SOM+ and MS are under the control of GABAergic nucleus incertus (NI) input that is also activated by the shock and regulates the fear expression.

To enable activity recordings from genetically defined interneuronal populations in the CA1 during CFC, Lovett-Barron et al. (2014) developed a variation of CFC for head-fixed mice (hf-CFC) that is compatible with two-photon Ca^2+^ imaging. They then established that, following US presentation, SOM+ interneurons (mainly the oriens-lacunosum moleculare (OLM) cells) are excited by cholinergic projections that arrive at the CA1 from the medial septum, which in turn leads to enhanced distal dendritic inhibition onto CA1 PCs and restricts the activation of these cells by foot-shock-evoked excitation (Figure 2). Subsequently, the inactivation of SOM+ interneurons during the US hindered the consolidation of contextual fear memory. Another interesting study reported that contextual fear conditioning resulted in the increased density of dendritic spines in SOM+ interneurons (Schmid et al., 2016), revealing structural plasticity that can be important for enhanced recruitment of these cells during fear memory acquisition. In addition, the consolidation of fear memory required activation of the mechanistic target of rapamycin complex-1 (mTORC1) pathway, which so far has been considered a key component in the regulation of protein synthesis and induction of the long-term memory (Tang et al., 2002; Costa-Mattioli et al., 2009). Importantly, interference with mTORC1 pathway in SOM+ interneurons resulted in impaired contextual fear memory (Artinian et al., 2019). Similarly, another recent study revealed that contextual fear conditioning was associated with a reduction in phosphorylation of eIF2α, an important regulatory element for the protein synthesis-dependent LTP in PCs and SOM+ interneurons (Sharma et al., 2020). As a result, the ablation of p-eIF2α in SOM+ cells enhanced fear memory by increasing protein synthesis and lowering the threshold for LTP induction. Moreover, SOM+ interneurons’ silencing immediately after the conditioning impaired fear memory (Sharma et al., 2020). Taken together, these studies indicate that encoding and consolidation of contextual fear memory in the CA1 is primarily controlled *via* activation of SOM+ interneurons that regulate burst firing of PCs and induction of long-term plasticity.

The current model furthermore suggests that the memory traces in the hippocampus are represented by a specific subset of PCs that make memory engrams because of enhanced intrinsic and synaptic excitability, which in turn can be actively regulated by specific subsets of inhibitory interneurons (reviewed in Josselyn and Tonegawa, 2020; Topolnik and Tamboli, 2022). For example, a recent tour-de-force study suggested that the non-engram cells are actively excluded from fear memory traces by dendritic inhibition provided by the SOM^+^ interneurons (Szőnyi et al., 2019). Moreover, this process can be tightly regulated by GABAergic projection from the brainstem nucleus incertus (NI), which targets specifically SOM+ cells as well as the excitatory inputs from the medial septum converging onto SOM+ cells (Szőnyi et al., 2019). Optogenetic activation of NI GABAergic neurons precisely at the moment of US impaired fear memory formation, whereas optogenetic inhibition during fear conditioning led to enhanced contextual memories, indicating that NI-to-SOM+ interneuron input plays a crucial role in regulating contextual fear memory acquisition.

The role of PV+ cells in contextual fear memory remains controversial, with different results reported depending on the memory induction paradigm and manipulation strategy (Lovett-Barron et al., 2014; Ognjanovski et al., 2017; Artinian et al., 2019; Khlaifia et al., 2022). For example, in a study conducted by Ognjanovski et al. (2017), the role of CA1 PV+ interneurons had been explored during single trial contextual fear memory consolidation using chronic stereotrode recordings of the activity of fast-spiking PV+ cells continuously for 48 h. As a result, they observed increased PV+ spike field coherence along with the principal cells during the first 6 h following CFC. Furthermore, chemogenetic inhibition of PV+ cells reduced fear, indicating that PV+ cells contribute to contextual fear memory consolidation. On the other hand, optogenetic silencing of CA1 PV+ cells during US (Lovett-Barron et al., 2014) or the conditional manipulations with eIF2α or Rptor signaling in CA1 PV+ cells had no effect on contextual fear memory consolidation (Khlaifia et al., 2022). In the CA3 hippocampal region, the activity of PV+ interneurons was required for contextual fear memory consolidation (Figure 2; Donato et al., 2013). Whereas the underlying circuit mechanisms are still poorly understood, they likely involved increased plasticity of PV+ interneuron networks. In particular, PV+ interneurons have been found in different states, depending on their level of activity (Donato et al., 2013). A low-PV network configuration has been associated with enhanced synaptic plasticity, memory consolidation, and retrieval, whereas a high-PV network configuration resulted in the impairment of these functions. Interestingly, the switch to a low-PV network configuration was associated with an increased innervation of PV+ interneurons by VIP+ cells. These findings reveal an important role of local circuit disinhibition in memory consolidation, and a network plasticity mechanism that involves VIP–PV microcircuit reconfiguration to control the functional state of PV+ interneurons during fear memory consolidation (Figure 2). Nonetheless, the so far controversial findings regarding the role of hippocampal PV+ interneurons in fear memory require further detailed examination using temporally precise manipulations at different stages of fear memory acquisition.

The hippocampal DG is another critical region in fear memory formation and consolidation (Kheirbek et al., 2013; Pierson et al., 2015). The early immediate gene c-Fos has been widely used for the evaluation of neuronal activity in this region owing to its transient and rapid upregulation upon experience (Smeyne et al., 1992; Reijmers et al., 2007). cFos expression in granule cells (GCs) was induced by the exploration of a new environment (Liu et al., 2012; Deng et al., 2013). It appears that exploration can be associated with the formation of an active neuronal ensemble that becomes both necessary and sufficient for representing the context mnemonically, and can be considered the cellular engram (Ramirez et al., 2013; Denny et al., 2014). Furthermore, contextual memory can be retrieved by reactivating the cFos-expressing neuronal ensembles in the fear conditioning paradigm (Tayler et al., 2013). The fear memory has been largely assigned to excitatory neurons and was associated with the activation of the CREB transcription factor, which modulates cellular excitability, ultimately determining memory allocation (Han et al., 2007; Stefanelli et al., 2016). Whereas the selection of neuronal ensembles appears to be governed by the cell autonomous mechanisms (Rogerson et al., 2014), local circuit mechanisms may also contribute to the formation of cellular engrams. Indeed, pharmacogenetic inactivation of DG SOM+ interneurons caused impairment in the acquisition of fear memory by forcing the recruitment of GCs (Stefanelli et al., 2016).

In addition to PV+ and SOM+ interneurons, VIP+ cells that engage in both inhibitory and disinhibitory circuit motifs in the hippocampus may be involved in fear memory control by regulating a balance between inhibition and disinhibition. These cells have been broadly categorized onto VIP+ basket cells (VIP-BCs) and VIP+ cells that inhibit other interneurons and are therefore considered as VIP+ interneuron-specific (IS) cells (Acsády et al., 1996; Chamberland et al., 2010; Tyan et al., 2014; Francavilla et al., 2018; Kullander and Topolnik, 2021). In the CA1 area, VIP-BCs co-express CCK and contact the somata of PCs, thus providing local inhibition. The CCK-BCs also contact PV+ BCs (Karson et al., 2009; Dudok et al., 2021), which may result in the disinhibition of PCs; thus, VIP-BCs may play a dual function. In contrast with VIP-BCs, other VIP+ interneurons may specialize in targeting only inhibitory interneurons, thus resulting in PC disinhibition (Acsády et al., 1996; Tyan et al., 2014; Francavilla et al., 2018); although, the connectivity motifs of different VIP+ subtypes remain to be established. In the CA1 hippocampal area, VIP+ IS interneurons can be further subdivided into three subtypes: type 2 IS cells (VIP-IS2), type 3 IS cells (VIP-IS3), and long-range projecting VIP+ cells (VIP-LRP; Acsády et al., 1996; Tyan et al., 2014; Francavilla et al., 2018). VIP-IS2 cells have somata located at the stratum radiatum/lacunosum moleculare border and form synapses with interneurons located within the stratum radiatum, including calbindin-positive and VIP+ cells (Acsády et al., 1996). In turn, VIP-IS3 cells co-express calretinin and have soma located in the stratum pyramidale or radiatum; moreover, they target interneurons located within the stratum oriens/alveus, mostly the SOM+ OLM cells that provide distal dendritic inhibition to PCs (Acsády et al., 1996; Tyan et al., 2014; Luo et al., 2020). VIP-LRP cells, as suggested by their name, can project from the CA1 region of the hippocampus to more distant areas, such as the subiculum (Francavilla et al., 2018). These cells can express the muscarinic receptor 2, calretinin, and enkephalin, and by their activity patterns *in vivo* correspond to theta-off cells, as they exhibit reduced firing during theta-run episodes but show increased activity during immobility (Francavilla et al., 2018; Luo et al., 2019). By employing the paired patch-clamp recording technique, it was observed that VIP-LRP cells do not contact the CA1 PCs and prefer different types of interneurons. The local preferential targets of VIP-LRP are the OLM cells, whereas in the subiculum, they establish contacts with interneurons as well as PCs, suggesting a region-specific function (Francavilla et al., 2018). As a population, VIP+ interneurons have been instrumental in regulating the goal-directed spatial learning (Turi et al., 2019). Furthermore, due to the expression of the alpha5 GABA_A_ receptor subunit at the IS3–OLM synapses, IS3 cells have been shown to control anxiety (Magnin et al., 2019). However, the functional role of VIP+ interneurons during fear learning remains to be determined.

## Animal studies: PFC and other neocortical regions

In addition to the amygdala and hippocampus, the PFC is critically involved in fear learning and extinction. It has been reported that the dorsal PFC supports fear expression, whereas the ventral PFC mediates fear extinction in both animal and human studies (Quirk et al., 2006; Sierra-Mercado et al., 2011; Milad and Quirk, 2012; Riga et al., 2014; Singh et al., 2018). Furthermore, the mPFC lesions revealed a significant role of this area in the extinction of cued fear memory (Morgan et al., 1993), which was further supported by pharmacological studies that assessed the roles of the PL-PFC vs. that of the IL-PFC. As such, the infusion of tetrodotoxin (Na^+^ channel blocker) and muscimol selectively into the PL-PFC followed by fear conditioning reduced both the cued and contextual fear expression (Laurent and Westbrook, 2009; Sierra-Mercado et al., 2011). Furthermore, electrical stimulation of the IL-PFC paired with a conditioned tone resulted in a decreased level of freezing, suggesting a role for the IL-PFC in fear extinction, in contrast with the PL-PFC, which promotes fear expression (Milad and Quirk, 2002; Milad et al., 2004). Taken together, these data highlight the region-specific roles of the PL-PFC and IL-PFC in fear learning and extinction.

**Figure 3:**
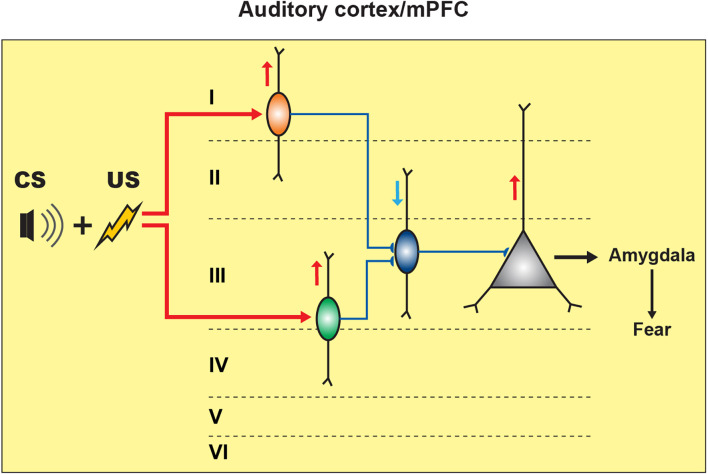
Inhibitory and disinhibitory circuit motifs in neocortical regions support fear memory formation. In the auditory cortex (A1), CS-US pairing elicits fear memory encoding by activating the L1 inhibitory neuronal population (orange) that can cause the disinhibition of L2/3 PCs by restricting the PV+ cell (blue) perisomatic inhibitory input onto the PCs, resulting in fear response expression. In the mPFC, CS-US association activates the SOM+ cells (green) that cause the disinhibition of PCs by inhibiting the PV+ cells’ activity, resulting in fear expression.

The primary auditory cortex (A1) is also involved in fear learning. The role of this region has been studied using the local administration of muscimol prior to fear conditioning, which resulted in a decrease in the fear-conditioned response (Banerjee et al., 2017). Single-unit recordings together with optogenetic and pharmacological manipulations in A1 revealed an important role of a disinhibitory circuit motif for associative learning (Letzkus et al., 2011). In this case, the aversive stimulus (US) activated the inhibitory interneurons located in layer 1 (L1) *via* cholinergic projections emerging from the basal forebrain region. As L1 interneurons inhibit PV+ basket cells, the US resulted in disinhibition of the layer 2/3 (L2/3) PCs, thus highlighting the circuit disinhibition as an important mechanism in associative learning (Figure 3; Letzkus et al., 2011). Across cortical regions, PV+ interneurons provide a powerful perisomatic inhibition to PCs (Pfeffer et al., 2013; Lee et al., 2014). Specifically, electrophysiological recordings in brain slices have revealed that PV+ interneurons located in the mPFC mediate feed-forward inhibition, which is crucial for maintaining the cortical excitation/inhibition balance (Lee et al., 2014). Moreover, the firing output of some PFC PV+ interneurons has been phase-locked to gamma oscillations (Kim et al., 2016). In addition, these cells regulate different types of cognitive behaviors and are affected by anxiety, panic attack, and depression (Sauer et al., 2015; Kim et al., 2016; Sauer and Bartos, 2022).

SOM+ interneurons provide inhibition to the apical dendrites of the L2/3 and layer 5 (L5) PCs. This population comprises Martinotti and non-Martinotti cell types that are distributed in layer 2 to layer 6 (Tremblay et al., 2016). The SOM+ interneurons with somata located within layer 4 (L4) and L5 receive input from thalamic projections (Naka and Adesnik, 2016). Interestingly, L2/3, L4, and L5 SOM+ interneurons receive complementary synaptic inputs to regulate the balance between top-down and bottom-up inputs (Naka et al., 2019). In the visual cortex, stimulation of a single L2/3 PC can trigger the activation of about 30% of SOM+ interneurons (Kwan and Dan, 2012). In the mouse barrel cortex, synaptic tracing using the rabies virus has demonstrated the cholinergic innervation of SOM+ cells, with presynaptic projection neurons located within the nucleus basalis of Meynert (Wall et al., 2016). In addition to neighboring PCs, SOM+ interneurons also receive input from distant brain areas (reviewed in Riedemann, 2019). The L5 non-Martinotti SOM+ cells do not inhibit L2/3 or L5 PCs; rather, they seem to contact L4 PCs (Riedemann, 2019). In addition, SOM+ cells can target VIP+ and PV+ interneurons (Pfeffer et al., 2013; Pi et al., 2013; Sohn et al., 2016). Moreover, although PCs are the primary postsynaptic target of superficial and infragranular SOM+ cells, fast-spiking interneurons are the main targets of L4 SOM+ cells (Xu et al., 2013).

In relation to fear learning, unfortunately, the roles of different types of neocortical interneurons remain largely unexplored. In L2/3 mPFC, the CS-US pairing resulted in increased spontaneous excitatory postsynaptic currents (sEPSCs) in SOM+ cells, rather than in PV+ interneurons (Cummings and Clem, 2020). These data echo the increased spine density in hippocampal CA1 SOM+ interneurons following CFC, suggesting that structural and functional plasticity of excitatory inputs to SOM+ cells may be a common sequel of fear conditioning across cortical regions. Furthermore, optogenetic activation of prefrontal SOM+ cells *in vivo* resulted in an increased freezing response. Surprisingly, in contrast to the amygdala and the hippocampus, combined optogenetic manipulations and electrophysiological recordings in brain slices revealed that prefrontal SOM+ interneurons cause disinhibition of PCs by eliciting the inhibition of PV+ interneurons (Figure 3; Cummings and Clem, 2020). Collectively, these data highlight a region-specific role of SOM+ interneurons.

Furthermore, the IL-PFC was involved in the suppression of fear memory *via* their excitatory projection to the BLA and GABAergic ITC clusters, which can limit the activation of the CeM output (Milad and Quirk, 2002). Moreover, the inhibitory effect of vHPC on IL-PFC PCs played a crucial role in the re-occurrence of extinguished fear. As such, activation of the vHPC projections to the IL-PFC could recruit IL-PFC PV+ interneurons and cause a relapse of extinguished fear (Marek et al., 2018). The inactivation of either the vHPC or BLA after fear learning was associated with an increase or a decrease in the firing of selective PL-PFC neurons (PCs or interneurons; Sotres-Bayon et al., 2012). The PCs exhibited a low firing rate after the inactivation of the BLA, suggesting that the BLA provides excitatory input to the PL-PFC, whereas vHPC inactivation resulted in a decreased firing rate in interneurons, indicating that the vHPC activates local interneurons to inhibit the firing of PL-PFC PCs *via* feed-forward inhibition (Sotres-Bayon et al., 2012). Thus, it can be concluded that during fear learning the vHPC gates the BLA input *via* PL-PFC PV+ interneurons (Sotres-Bayon et al., 2012).

Finally, during fear learning, the US can activate frontal VIP+ cells, thereby promoting the disinhibition of PCs necessary to facilitate the processing of the noxious signals. In this scenario, the US presentation can limit the activation of PV+ and SOM+ interneurons *via* selective recruitment of the VIP+ cells, therefore resulting in the perisomatic and dendritic disinhibition of PCs (Lee et al., 2013; Pi et al., 2013; but see Garcia-Junco-Clemente et al., 2017 for direct inhibition of PCs exerted by VIP+ cells during arousal in addition to disinhibition, which results in push-pull frontal circuit). Given that SOM+ cells are the primary targets of VIP+ interneurons in the neocortex, the resulting dendritic disinhibition of PCs during US is likely a dominating mechanism operating in cortical circuits during associative fear learning (Lee et al., 2013; Pfeffer et al., 2013; Pi et al., 2013). Furthermore, given the SOM-to-PV connectivity motif, the VIP+ input to SOM+ interneurons may also result in disinhibition of PV+ cells and gradual recovery of perysomatic inhibition of PCs during fear conditioning. Therefore, prefrontal VIP+ interneurons are well positioned to control the timing of perisomatic inhibition and dendritic disinhibition, which may be necessary for induction of associative plasticity but also for synchronous firing and cell assignment into functional ensembles.

## Conclusion and future perspectives

Although the data available thus far provide important insights regarding the structure and function of the neuronal circuits involved in fear memory, our understanding of how behavioral learning is implemented at the network level remains still limited. Several points need to be addressed to understand the mechanisms that contribute to the formation and regulation of fear circuitry. In particular, the extinction of fear memory that involves both the erasure and extinction-induced inhibition within fear circuits and allows to regulate the stability of fear memories (Herry et al., 2008) requires further investigation. How this phenomenon depends on the timing of extinction training, animal age, and sex, and specific connectivity motifs within threat circuitry still needs to be understood. Considering extinction as an important instrument in PTSD therapy (Milad and Quirk, 2012), it would be critical to explore further in human studies how the observed changes in structure and function of fear circuits affect the extinction paradigm. Likewise, in the animal models, it needs to be determined how the reduction in the volume of the amygdala, hippocampus, and PFC associated with PTSD correlates with functional deficits within specific neuronal populations that are involved in fear extinction, and how their function can be rescued in order to implement the efficient extinction paradigm in affected individuals.

In this regard, over the last few decades, the field has moved to exploring the contributions of different neuronal populations to animal behavior, thanks to rapidly developing gene targeting technologies. An increasing number of studies have investigated the functional role of inhibitory interneurons in different memory paradigms thereby revealing that interneurons are important players in diverse learning tasks. In relation to fear learning, these cells not only shaped the circuit response during fear conditioning but also showed changes in their properties during fear consolidation. Therefore, across different fear circuits, the network function appears to be tightly regulated *via* inhibitory circuit mechanisms. The PV+, SOM+ and VIP+ interneurons in the amygdala received more attention than in the other fear circuits. In BLA, perisomatic inhibition provided to PCs by the PV+ cells, and likely VIP-BCs (Rhomberg et al., 2018), during CS is followed by disinhibition during US because of VIP+ to PV+, and SOM+ connections (Wolff et al., 2014; Krabbe et al., 2019). Manipulating with these cell types impairs fear learning, indicating that they are all important for tight regulation of PC activity and induction of associative plasticity and memory to shape the amygdala fear response. Much less is known regarding the role of PV+ and VIP+ cells in the hippocampus, in particular in relation to CFC and extinction paradigms. Besides this, the role of these cell types in the prefrontal areas in fear learning, and most importantly in fear extinction, also needs to be discovered to have a complete understanding of the inhibitory components of the circuitry responsible for processing and regulation of fear memory.

Circuit disinhibition has recently emerged as an important mechanism that participates widely in memory-related paradigms by limiting the firing of the GABAergic interneuronal populations and providing additional ways for the modulation of network activity. Disinhibition phenomenon appears to be critical in fear conditioning and related long-term changes that take place in different neural circuits (Letzkus et al., 2011). So far, distinct behavioral phenomena, such as auditory fear conditioning and spatial navigation, were causally linked to disinhibition in different compartments of PCs in several cortical and limbic areas, and at time scales ranging from milliseconds to days (Sparta et al., 2014; Kim et al., 2016; Cummings and Clem, 2020; Dudok et al., 2021), suggesting that disinhibition is a conserved circuit mechanism that is required for learning and memory to occur. However, further studies are necessary to determine whether similar disinhibitory patterns exist in different circuits. In addition, abnormal disinhibition may impair memory performance and result in disruptive consequences (McGarrity et al., 2017).

In summary, a working model of fear learning can be proposed in which traumatic events recruit specific interneuronal populations that control PCs and other interneurons to create the representative PC ensembles, or memory engrams, *via* the phenomena of inhibition and disinhibition, respectively. As such, whereas some PCs are excluded from memory engrams because of active inhibition, the others become linked to engrams because of increased intrinsic and synaptic excitability facilitated by disinhibition. The changes in inhibitory connections can also shift the network balance, thus altering the coding of the fear experience. Therefore, inhibitory neuronal populations responsible for local circuit inhibition and disinhibition are currently considered as a powerful component in the creation of mental fear representations. Further studies are required to establish the role of specific inhibitory and disinhibitory patterns in complex fear-related behavioral adaptations and a direct link between a stressful event and the functional plasticity of specific circuit elements.

## Author contributions

SS wrote the first version of the manuscript and prepared the figures. LT prepared the final version of the manuscript. All authors contributed to the article and approved the submitted version.
